# Supplementary material for: ATF4 leads to glaucoma by promoting protein synthesis and ER client protein load
Source: Nat Commun. 2020 Nov 5;11:5594. doi: 10.1038/s41467-020-19352-1 (PMC7644693; doi:10.1038/s41467-020-19352-1)
Supplement: Supplementary file 1 — Supplementary Information [file 41467_2020_19352_MOESM1_ESM.pdf]

Supplementary figures

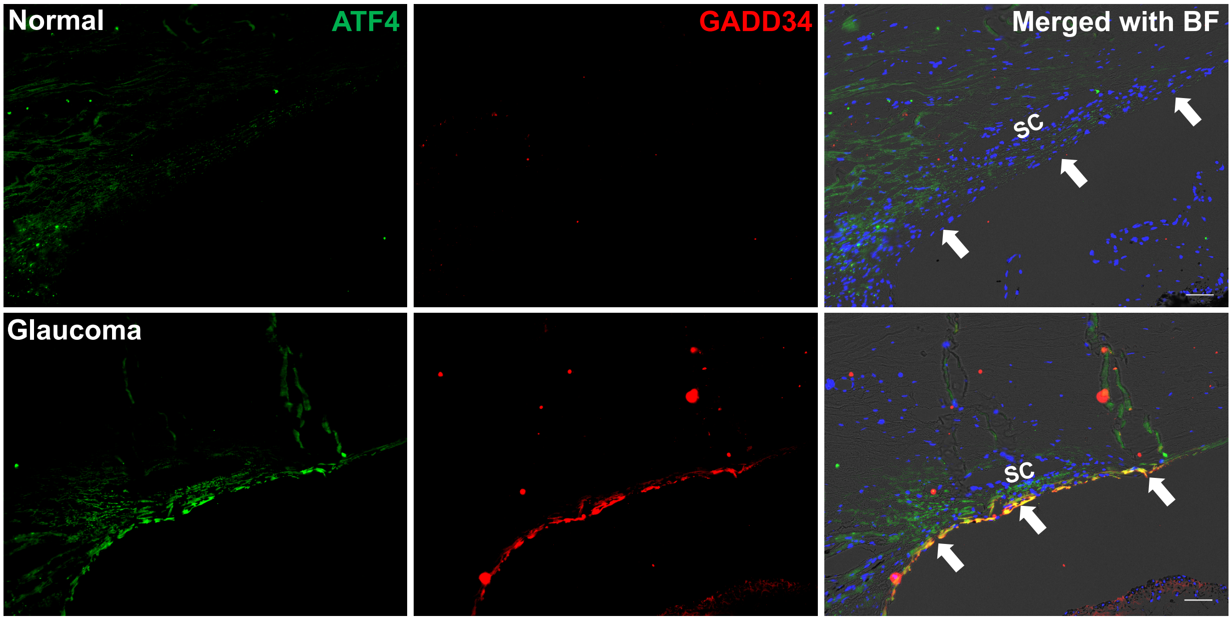

SI.1: Increased ATF4 and GADD34 staining in glaucomatous human TM tissues. Representative low magnification (200x) images of ATF4 and GADD34 immunostaining are shown to demonstrate overall pattern of immunostaining in the entire TM region. N=9 and n=11 biologically independent samples from normal and glaucoma groups respectively. Arrows represent TM region; SC= Schlemm's canal; scale bar is 50µm.

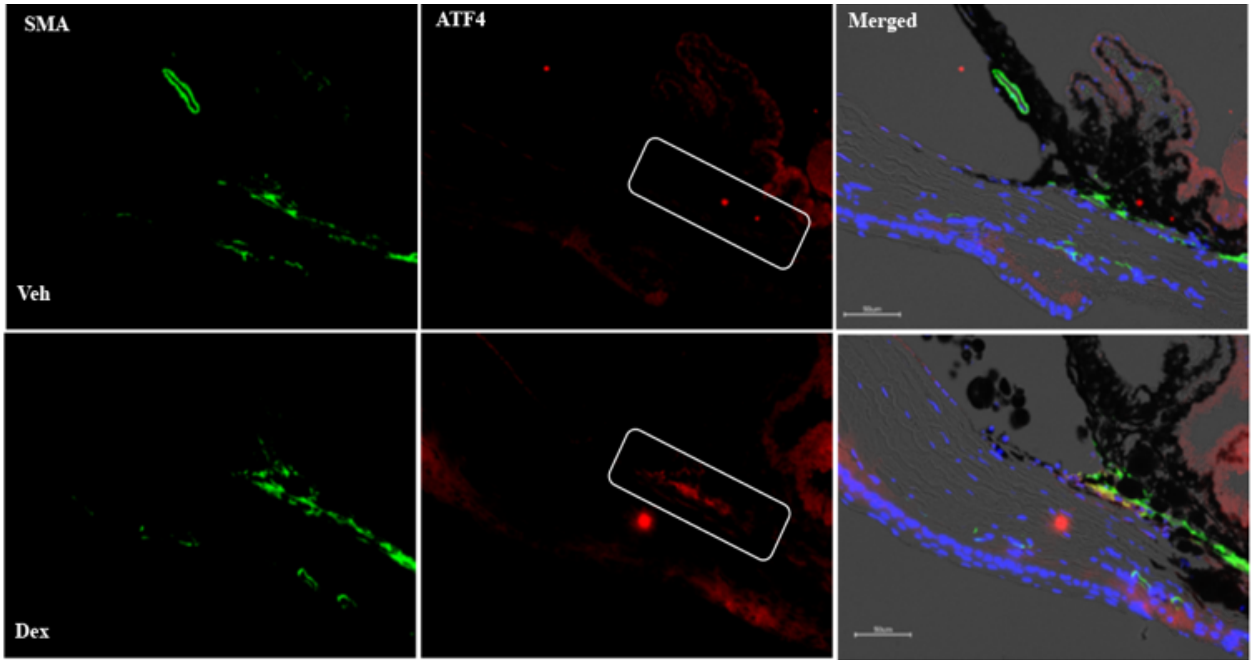

SI.2: Increased ATF4 and SMA in the TM of mouse model of Dex-induced ocular hypertension. Immunostaining for α-smooth muscle actin (α-SMA) and ATF4 was performed in anterior segment tissues from 5-weeks vehicle or Dex-injected eyes. Increased α-SMA and ATF4 staining was observed in TM region of Dex-treated eyes compared to vehicle. N=3 biologically independent samples. White box represents TM region; scale bar is 50μm.

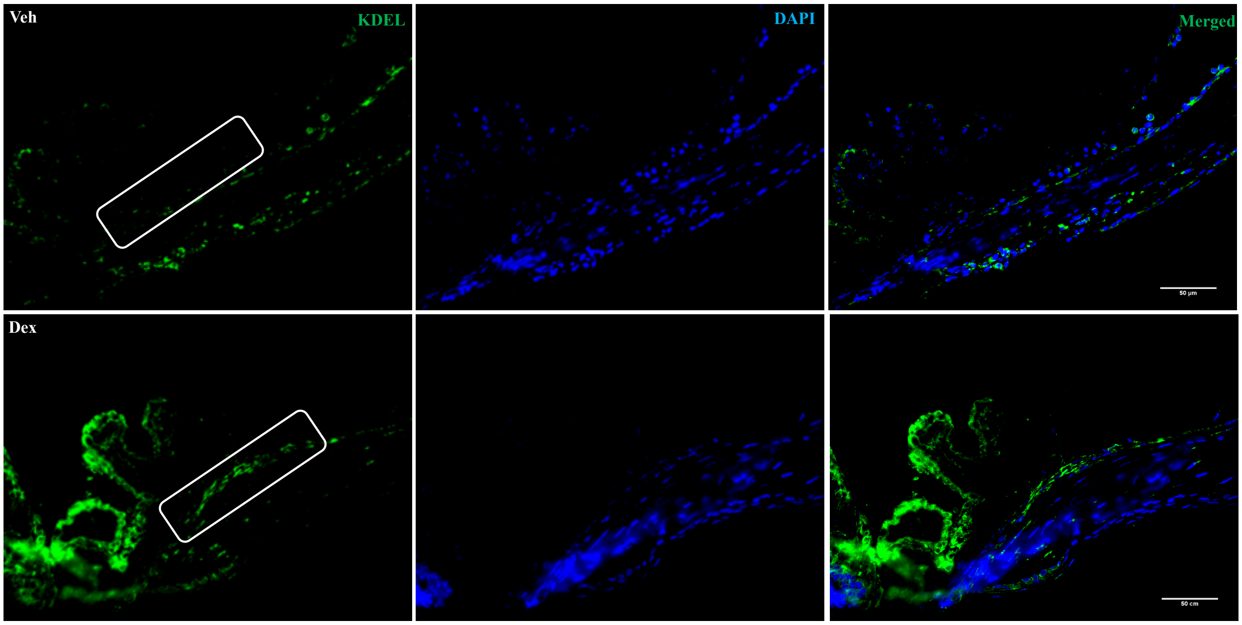

SI.3: Increased KDEL in the TM of Dex-treated mice. Immunostaining for KDEL (ER stress marker) in mice injected with vehicle or Dex for 5-weeks via periocular route. Increased KDEL staining in the TM region (white box) was observed in Dex-treated mice compared to vehicle-treated mice (n=3 each group). TM region indicated by white box and scale bar is 50μm.

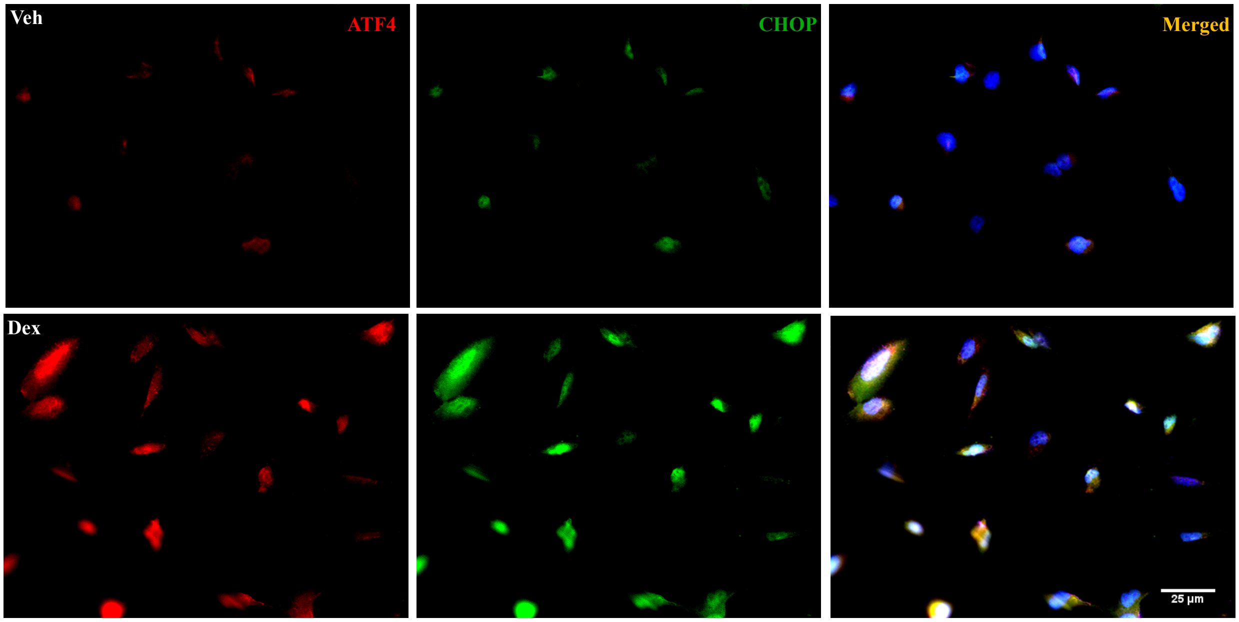

SI.4: Increased ATF4 and CHOP in Dex-treated GTM3 cells. GTM3 cells treated with vehicle (0.1% ethanol) and Dex (100nM) for 3 days. GTM3 cells were fixed and stained for ATF4 and CHOP. Dexamethasone treatment increased ATF4 and CHOP staining compared to the vehicle treatment (n=2 independent experiments). Most of ATF4 and CHOP was localized to nucleus suggesting transcriptional activity. Scale bar is 25μm.

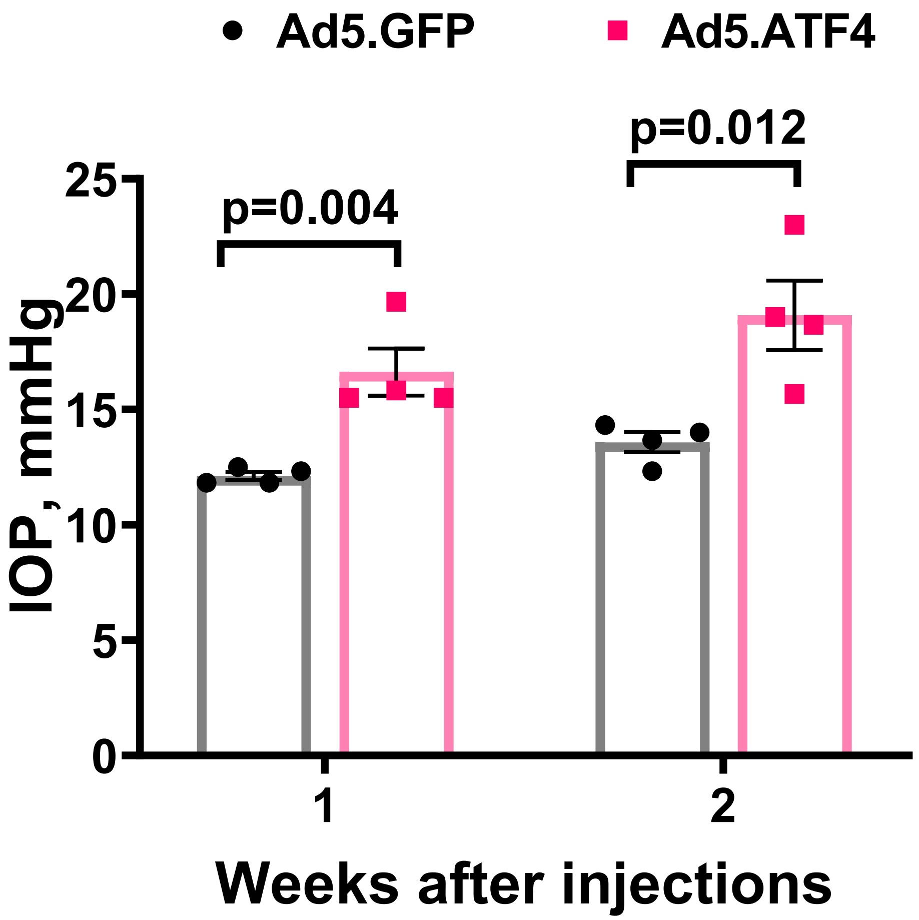

SI.5: Overexpression of GFP does not alter IOP significantly. C57 mice were injected intravitreally with Ad5.GFP (2×10^7 pfu/eye) or Ad5.ATF4 (2×10^7 pfu/eye) and IOPs were monitored weekly. While Ad5.ATF4 elevated IOP significantly, Ad5.GFP did not alter IOP. These findings indicate that expression of GFP does not elevate IOP significantly. N=4 biologically independent samples, data are presented as mean ± SEM, multiple t tests using holm-sidak method.

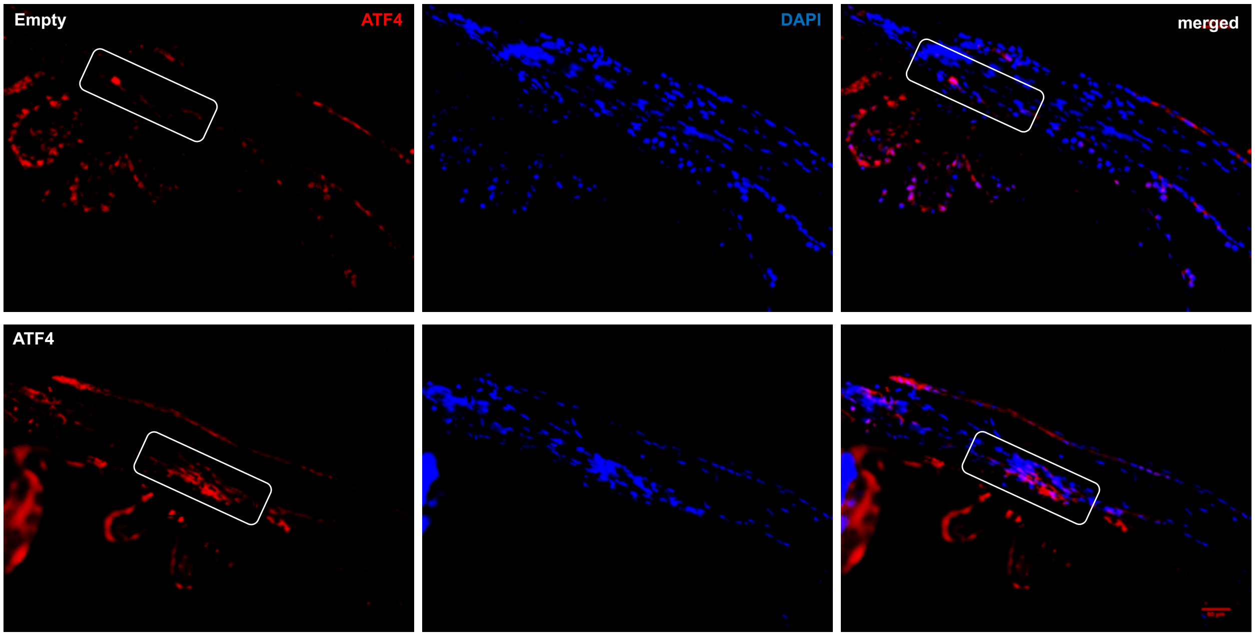

SI.6: Expression of ATF4 in TM tissues of Ad5.ATF4-injected mice. C57BL/6J mice were injected intravitreally with Ad5.Empty or Ad5.ATF4 ($2x10^7$ pfu/eye). Six weeks after injections, the eyes were enucleated, fixed, and paraffin sections were immunostained with ATF4. Increased ATF4 staining was observed in TM region of Ad5. ATF4 injected eyes (n=3 biologically independent samples from each group). White box shows the TM region. Scale bar is 50 µm.

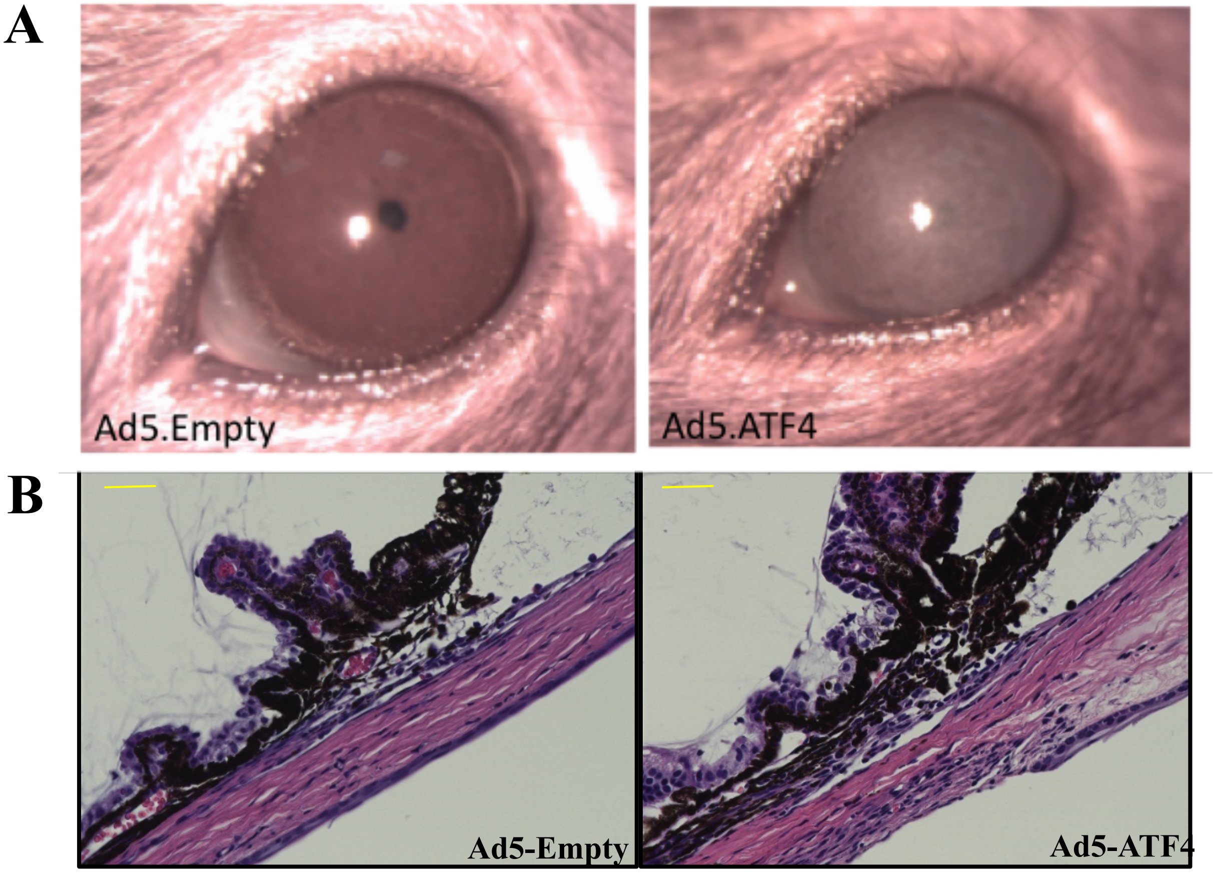

SI.7: Ocular inflammation in adenoviral injected eyes. **A**) Slit lamp images of Ad5 injected mice shows mild inflammation in Ad5.empty injected mice and moderate inflammation in Ad5.ATF4 injected mice. **B**) Anterior segment tissues from Ad5. Empty or ATF4 injected mice was stained with H&E. ATF4 injected eyes showed corneal edema compared to Ad5-Empty injected mouse eyes. No obvious abnormalities observed at the TM in both groups and iridocorneal angle was open in both groups (n=3 biologically independent samples in each group). Scale bar is 50 μm.

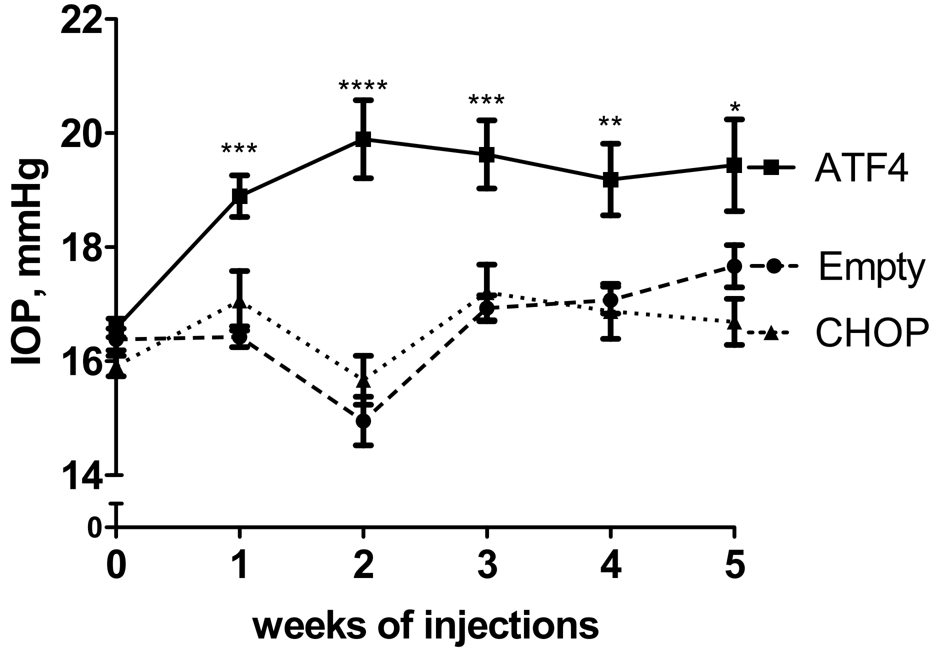

SI.8: Expression of ATF4 but not CHOP elevates IOP significantly in mice. C57BL/6J mice were injected intravitreally with lentiviral particles (2x10^6 TU/eye) containing GFP (control), ATF4 and CHOP. Daytime IOPs were monitored every week for 5 weeks using rebound tonometry. A significant IOP elevation was observed in ATF4 injected mice compared to the Empty and CHOP injected mice. CHOP injections did not alter IOPs and their IOPs were similar to empty injected mice. (n=8 in each group; data are presented as mean ± SEM, 2-way ANOVA, *$^*P<0.05$, $^{**}P<0.01$, $^{***}P<0.001$, $^{****}P<0.0001$*)

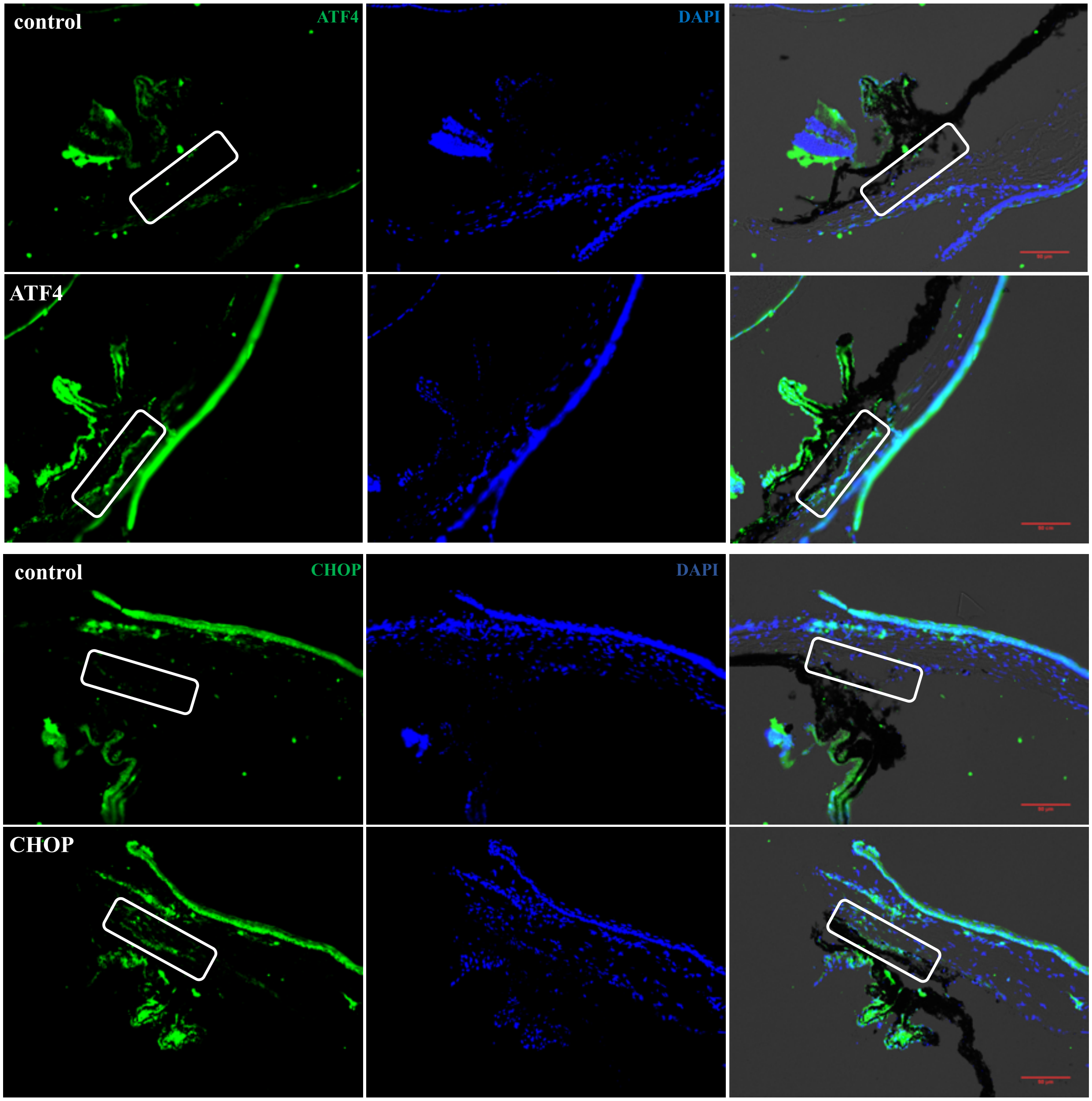

SI.9: Expression of ATF4 and CHOP in the TM of lentiviral injected mice. C57BL/6J mice were injected intravitreally with lentiviral particles expressing GFP, ATF4 or CHOP. 10-weeks after injections, anterior segment tissues were stained with ATF4 (Top panel) and CHOP (Bottom panel). As shown in white square box representing TM region, increased ATF4 and CHOP staining was observed in ATF4 and CHOP injected mice respectively (n=4 from each group). Scale bar is 50 µm.

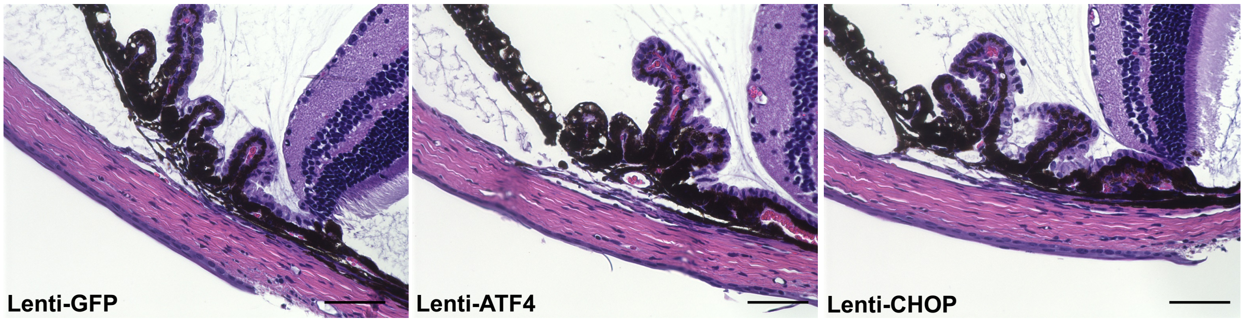

SI.10: Lentiviral injections of GFP, ATF4 and CHOP does not lead to ocular inflammation in mice. Anterior segment tissues stained with H & E revealed that lentiviral particles expressing GFP, ATF4 or CHOP does not cause ocular abnormalities. Iridocorneal angle is open and no inflammatory cells were present in outflow pathway in these mice. In addition, unlike Ad5 injections, lentiviral particles did not cause corneal edema (n=4 biologically independent samples; Scale bar is 50 μm).

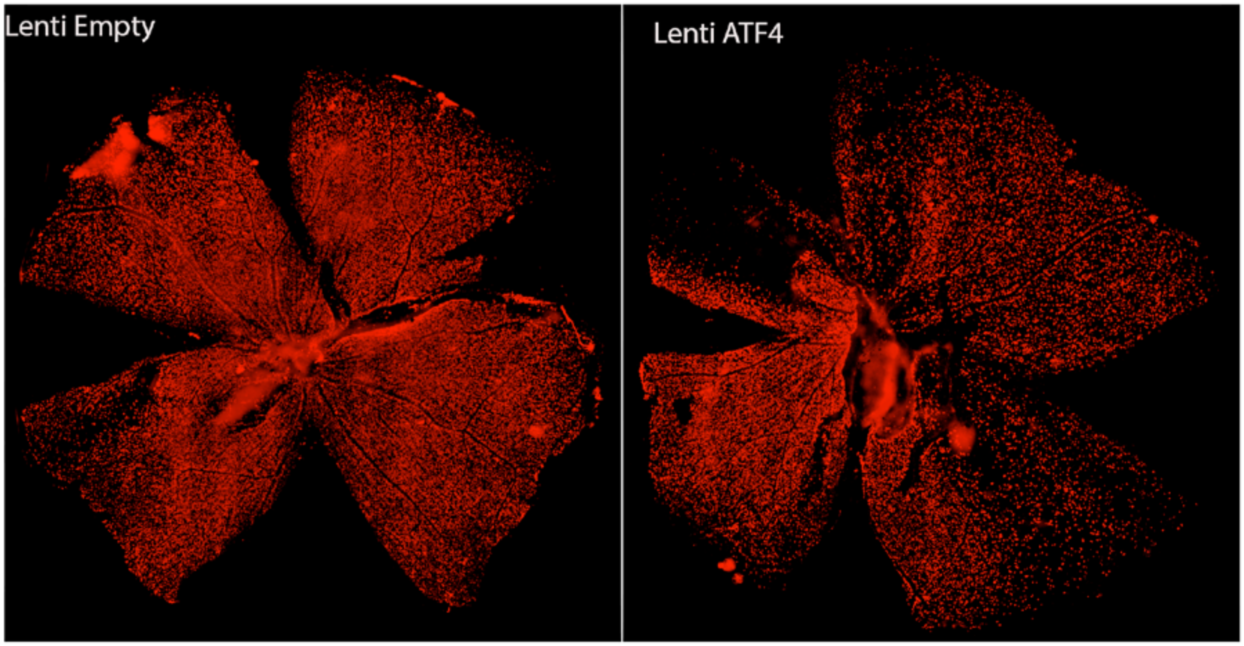

SI.11: Representative images of whole mount RBPMS labeling of RGCs. RBPMS (RGC marker) staining of whole mount retinas from the mice collected 10-weeks after administering lentiviral injections of GFP (Lenti Empty) and ATF4. ATF4 injected retina showed loss of RGCs. (n=7 GFP injected group and n=8 ATF4 injected group).

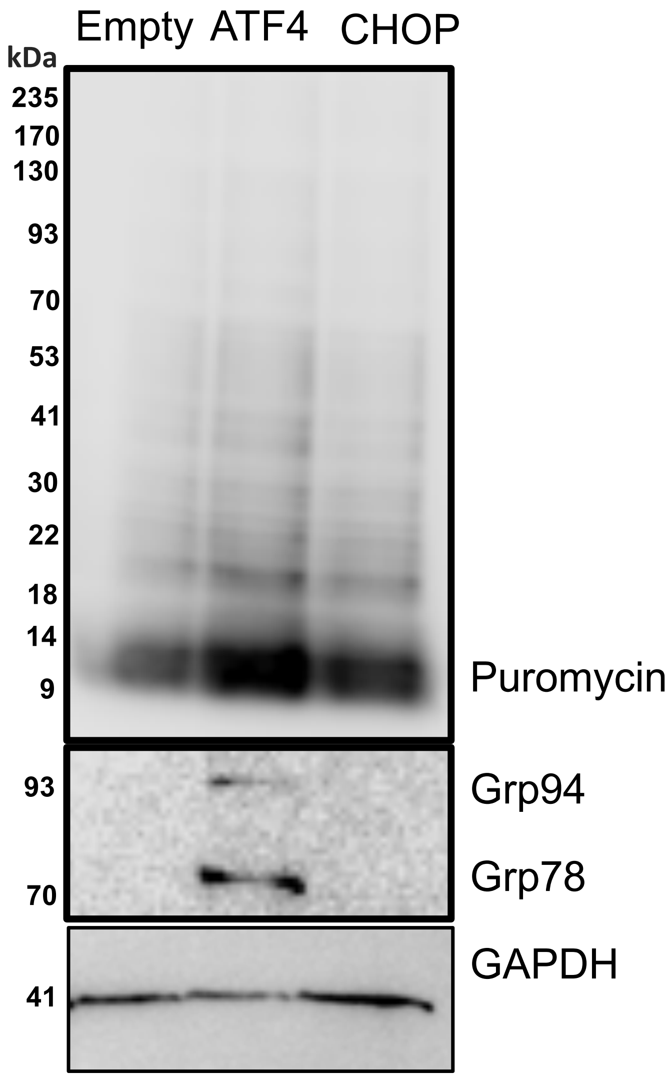

SI.12: Expression of ATF4 leads to increased ER client protein load in TM cells. GTM3 cells transfected with ATF4 or CHOP were incubated with puromycin (10µg/ml) for 30 minutes before harvesting cell lysates. Total ER fraction was isolated and equal amount of ER lysates were subjected to Western blot analysis using anti-puromycin, GAPDH and KDEL antibodies. Increased puromycin incorporation observed in the total ER fractions of ATF4 transfected cells (n=2 independent experiments) signifying a higher rate of *de novo* protein synthesis of secretory proteins.

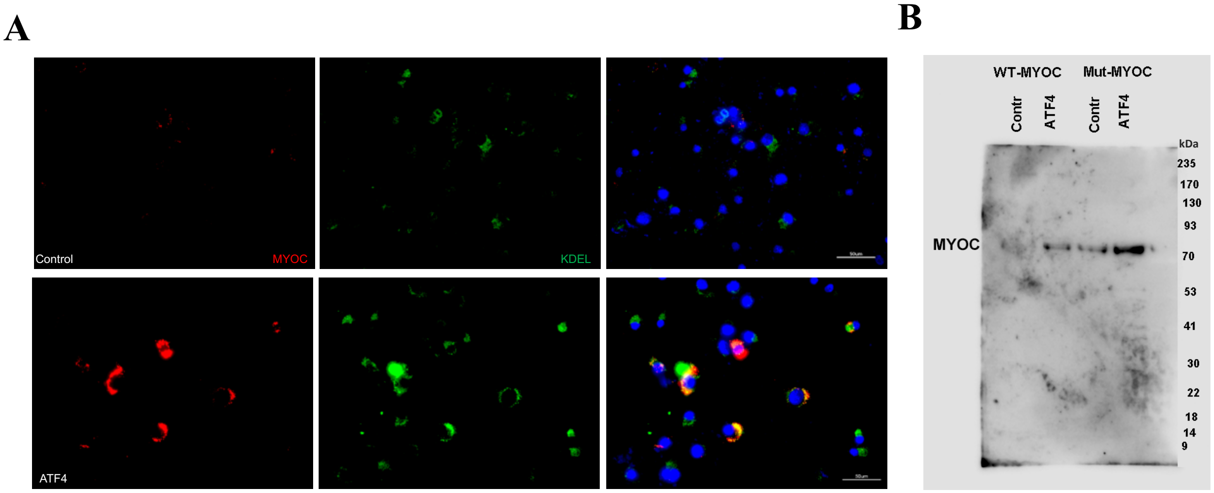

SI.13: Expression of ATF4 leads to accumulation of WT and mutant myocilin and induces ER stress in GTM3 cells. GTM3 cells stably expressing WT or mutant (Y437H) myocilin tagged with DsRed were transduced with lentiviral vectors expressing Empty and ATF4 for 3 days. **A**) Cells were fixed and stained for KDEL (ER stress marker). Increased intracellular myocilin and KDEL staining was observed in ATF4 transduced cells. A prominent co-localization of myocilin and KDEL in ATF4 transduced cells indicates WT myocilin accumulates intracellularly and induces ER stress in TM cells (n=2 independent experiments). Scale bar is 50µm. **B**) Triton insoluble pellets from GTM3 cells stably expressing WT or mutant myocilin with or without ATF4 were subjected to Western blot analysis for myocilin. Little or no WT myocilin was observed in control GTM3 cells expressing WT myocilin (First lane). As expected, GTM3 cells stably expressing mutant myocilin demonstrated increased level of triton insoluble myocilin (lane 3). Expression of ATF4 increased insoluble WT myocilin similar to mutant myocilin levels (lane 2). In addition, expression of ATF4 further exacerbated myocilin accumulation in GTM3 cells stably expressing mutant myocilin (lane 4). These data indicate that expression of ATF4 increases triton insoluble WT and mutant myocilin in GTM3 cells. N=2 independent experiments.

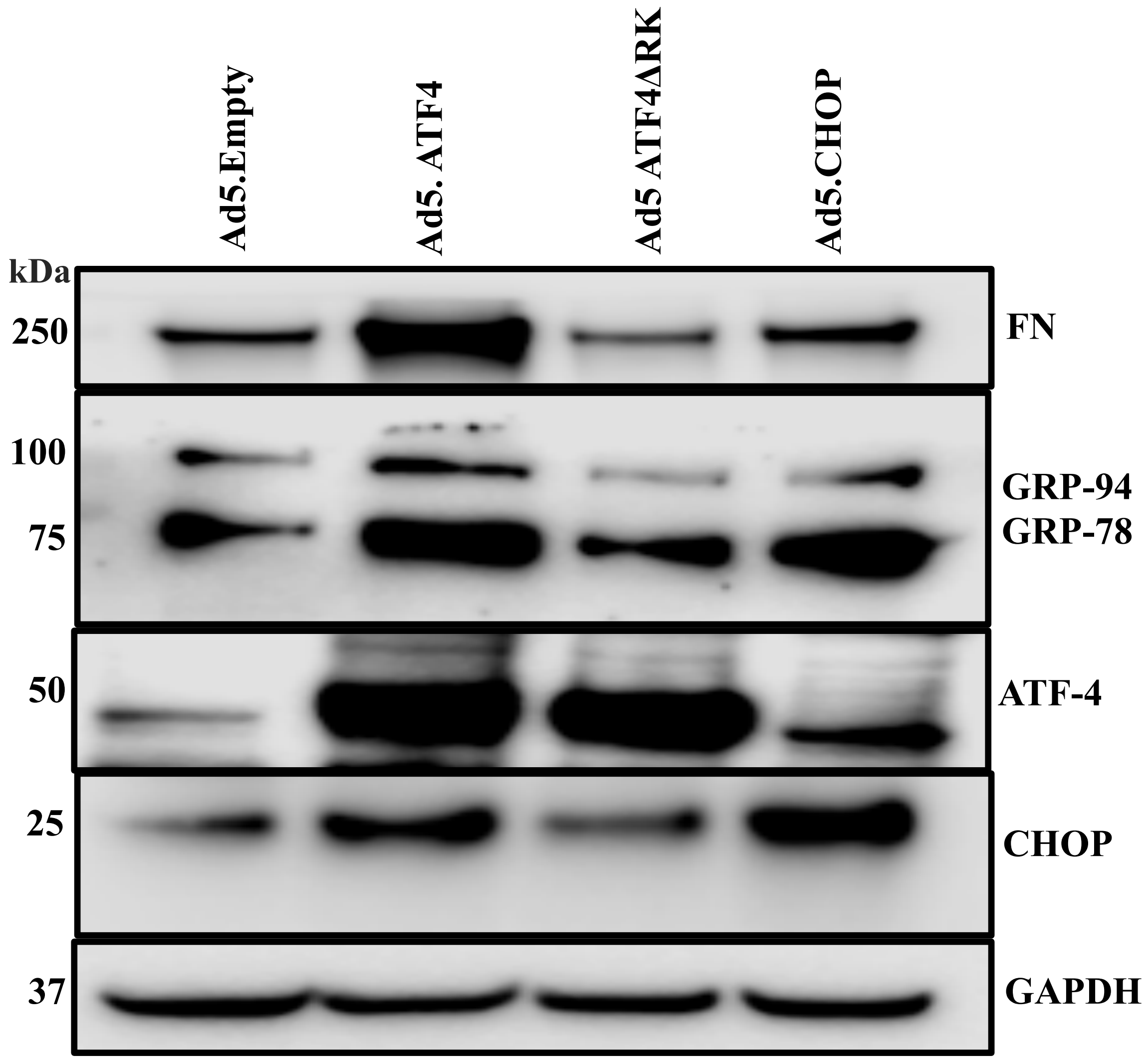

SI.14: Expression of ATF4 leads to increased ECM synthesis and ER stress in primary human TM cells. Primary human TM cells (n=3 cell strains) were transduced with Ad5.Empty, Ad5.ATF4, Ad5.ATF4ΔRK (a dominant negative inhibitor of ATF4) and Ad5.CHOP for 3 days. Cellular lysates were subjected to Western blot analysis for ECM (fibronectin) and ER stress (ATF4, CHOP, GRP78 &GRP94) markers. ATF4 led to increased ECM accumulation and ER stress while expression of CHOP or Ad5.ATF4ΔRK did not alter ECM and ER stress markers.

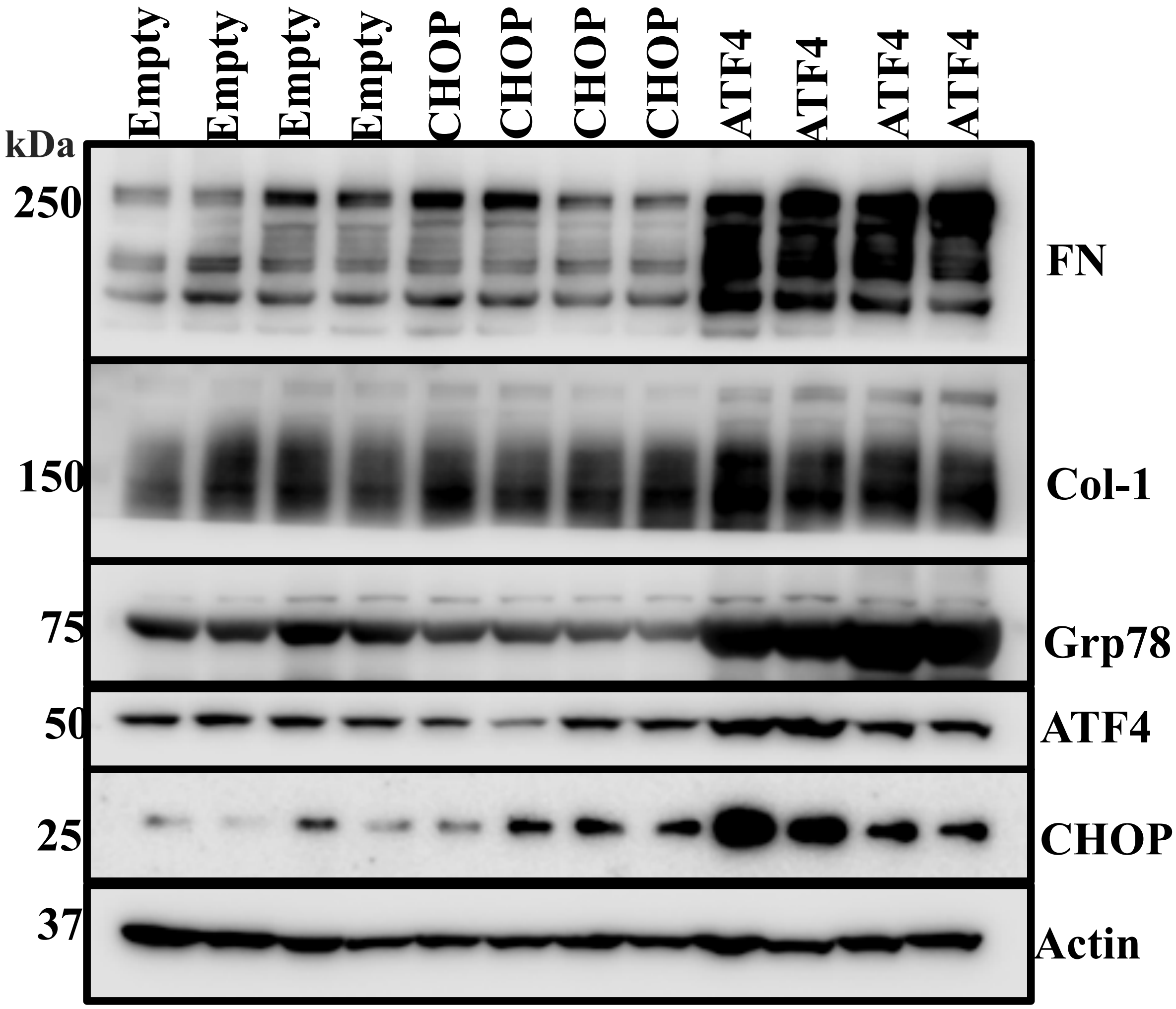

SI.15: Expression of ATF4 but not CHOP leads to induction of ER stress in mouse TM tissues. 3-months old C57BL/6J mice were intravitreally injected with Ad5.Empty, Ad5.ATF4 and Ad5.CHOP (2x10^7 pfu/eye). After 3-weeks of injections, anterior segment lysates were subjected to Western blot analyses of ECM (fibronectin, collagen-1) and ER stress (Grp78, ATF4 & CHOP) markers. ATF4 increased ECM and ER stress markers. N=4 biologically independent samples.

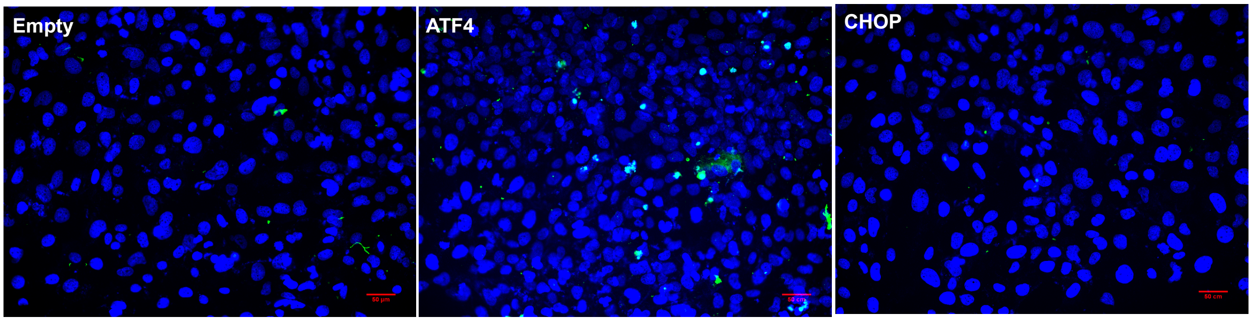

SI.16: Expression of ATF4 but not CHOP leads to TM cell death in NTM5 cells. NTM5 cells (n=3 replicates) were transduced with Ad5.Empty, ATF4 or CHOP for 36 hrs and fixed cells were analyzed by TUNEL assay. Increased number of TUNEL positive cells (green) were observed in Ad5.ATF4 transduced TM cells (n=3 independent experiments). scale bar is 50μm.

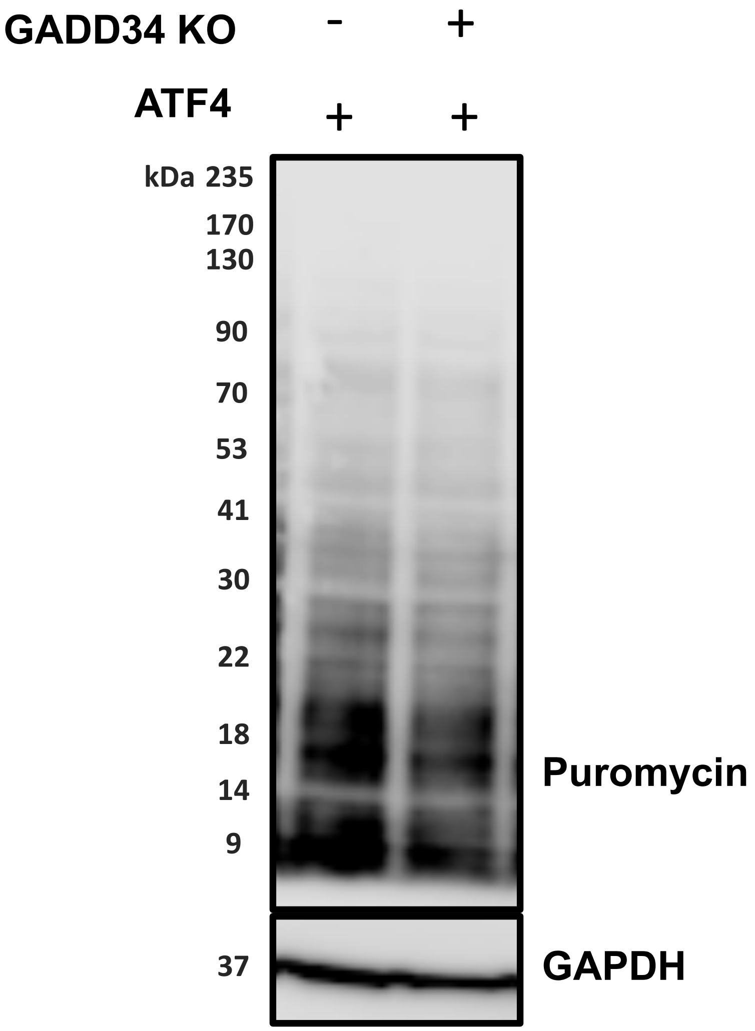

SI.17: Depletion of GADD34 reduces ATF4-induced de novo protein synthesis in GTM3 cells. GTM3 cells expressing ATF4 were transfected with plasmid expressing CRISPR-Cas9 targeting GADD34. Puromycin (10µg/ml) was added to cells for 30 minutes before harvesting cell lysates. Total cellular lysates were subjected to Western blot analysis using anti-puromycin and GAPDH antibodies. GADD34 knock down significantly reduces ATF4-induced protein synthesis. N=4 independent experiments.

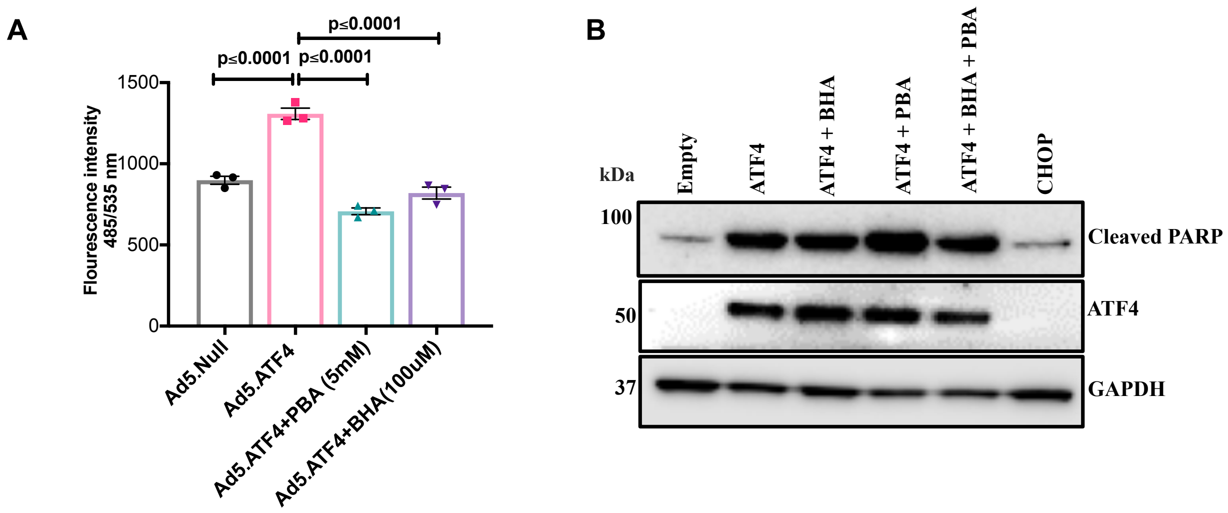

SI.18: ATF4 induces oxidative stress and inhibition of oxidative stress does not protect TM cells from ATF4-induced TM cell death. A) GTM3 cells transduced with adenoviral expression vectors containing Empty, ATF4 and treated with PBA (a chemical chaperone) and BHA (an antioxidant) for 2 days. Cellular reactive oxygen species (ROS) were measured by DCFDA assay. Adenoviral expression of ATF4 significantly increased the ROS production. PBA and BHA treatments inhibited ATF4 induced ROS production (n=3 independent experiments; data are presented as mean ± SEM, 1-way ANOVA. **B**) GTM3 cells were transduced with Ad5.Empty, Ad5.ATF4 and Ad5.CHOP and treated with PBA and BHA for 2 days. Cellular lysates were used for Western blot analysis for cleaved PARP (n=2 independent experiments). Expression of ATF4 increased cleaved PARP. However, treatment with BHA or PBA did not reduce ATF4-induced cleaved PARP further suggesting that reduction of oxidative stress does not prevent ATF4-induced cell death.

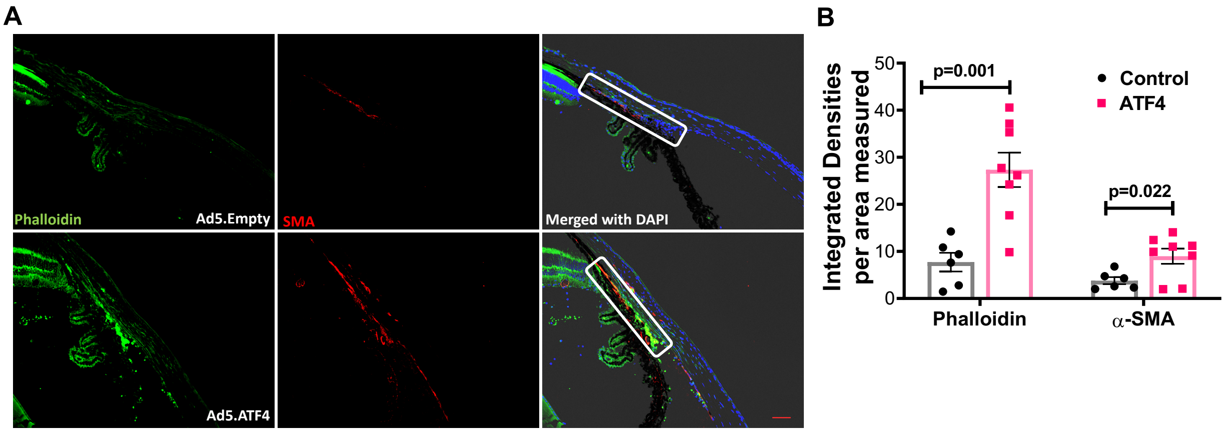

SI.19: ATF4 increases phalloiden and α-smooth muscle actin (αSMA) staining in mouse TM tissue. Since actin filaments and SMA are thought to regulate mechanical and contractile properties of TM[1-3], we further explored whether ATF4 alters actin and SMA in mouse TM. **A-B)** C57BL/6J mice were intravitreally injected with Ad5.empty or ATF4 for 2 weeks and anterior segments were stained with phalloidin and **α**SMA **(A)** (n=8 biologically independent samples from each group; Scale bar is 50 µm). The integrated densities of phalloidin and **α**SMA staining in the TM region were analyzed using image J **(B)**. ATF4 increased F-actin and SMA in mouse TM tissues indicating that ATF4 may reduce contractile functions of TM, increasing outflow resistance and IOP elevation. N=8 biologically independent samples; data are presented as mean ± SEM, 2-tailed unpaired t-test.

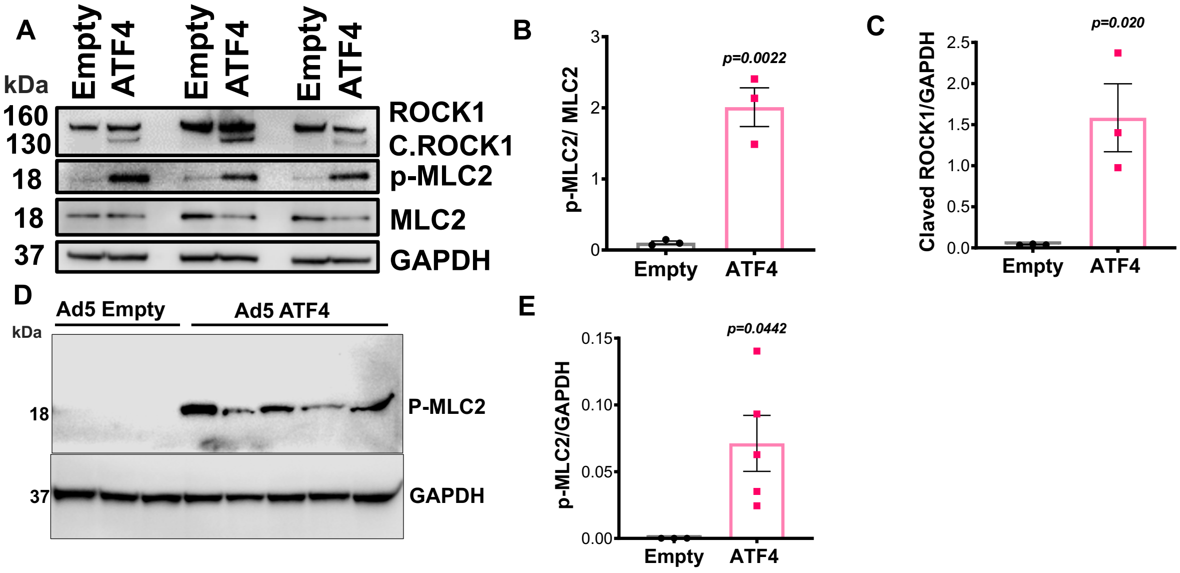

SI.20: ATF4 induces cleaved ROCK1 and phosphorylation of MLC2 in NTM5 cells and mouse anterior segment tissues. Rho/Rho-kinase pathway-mediated MLC phosphorylation is thought to modulate TM contractile function and regulate TM stiffness[1,4]. Activated (GTP-bound) Rho interacts with ROCK and activates its kinase activity, which phosphorylates MLC directly. The phosphorylation of MLC leads to rearrangement of cytoskeleton and stress fiber formation[1]. We examined whether ATF4 induces Rho-kinase pathway. NTM5 (n=3 technical replicates) were transduced with Ad5.Empty or ATF for 48 hours and cell lysates were subjected to Western blot (**A**) and its densitometric analyses (**B-C**) of cleaved ROCK1 and phosphorylated and total MLC2. ATF4 induced cleaved ROCK1 and increased pMLC2 in NTM5 cells (n=3 technical replicates, data are presented as mean ± SEM, 2-tailed unpaired t-test). Western blot (**D**) and its densitometric analyses (**E**) further demonstrated that ATF4 increases phosphorylation of MLC2 in mouse anterior segment tissues (n=3 from Ad5.Empty and n=5 from Ad5.ATF4 biologically independent samples, data are presented as mean ± SEM, 2-tailed unpaired t-test).

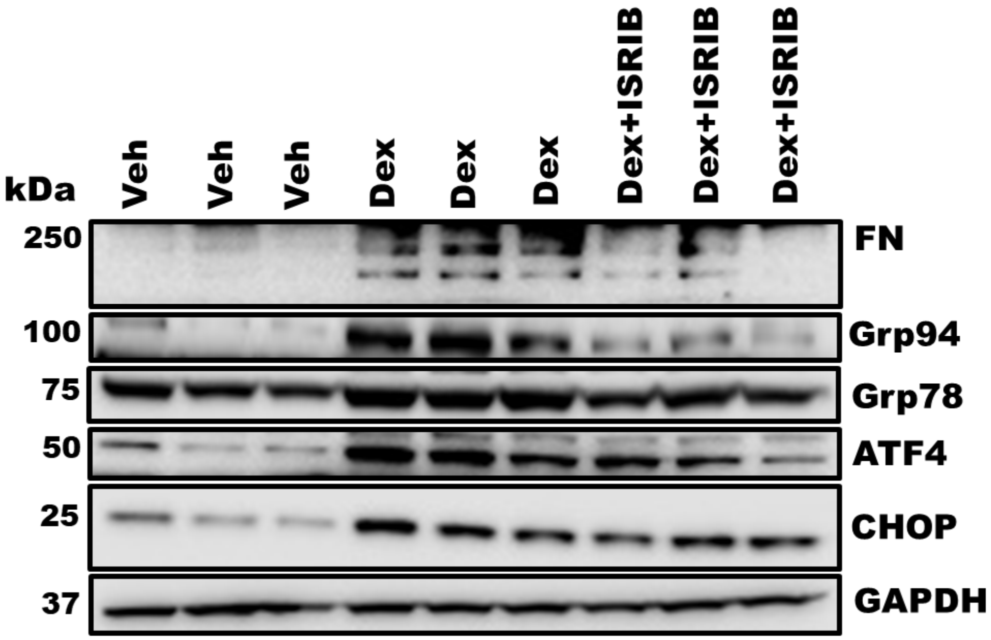

SI.21: ISRIB reduces Dex-induced fibronectin and ER stress in mouse TM tissues. C57 mice were injected with vehicle and Dex weekly via periocular conjunctival fornix injections. After 2-weeks, left eyes received 5 µl of 2mM ISRIB topical eye drops while the contralateral right eyes received vehicle eye drops (DMSO) twice daily. Western blot analysis of mouse anterior segment lysates for ECM and ER stress markers was performed. ISRIB treatment prevented Dex-induced FN and ER stress markers (n=3 biologically independent samples).

References:

1 Pattabiraman, P. P. & Rao, P. V. Mechanistic basis of Rho GTPase-induced extracellular matrix synthesis in trabecular meshwork cells. *Am J Physiol Cell Physiol* **298**, C749-763, doi:10.1152/ajpcell.00317.2009 (2010).

2 Peterson, J. A. *et al.* Latrunculin-A increases outflow facility in the monkey. *Invest Ophthalmol Vis Sci* **40**, 931-941 (1999).

3 Cai, S. *et al.* Effect of latrunculin-A on morphology and actin-associated adhesions of cultured human trabecular meshwork cells. *Mol Vis* **6**, 132-143 (2000).

4 Rao, P. V., Deng, P. F., Kumar, J. & Epstein, D. L. Modulation of aqueous humor outflow facility by the Rho kinase-specific inhibitor Y-27632. *Invest Ophthalmol Vis Sci* **42**, 1029-1037 (2001).
